# Advancing Public Health Using Regulatory Science to Enhance Development and Regulation of Medical Products: Food and Drug Administration Research at the Center for Biologics Evaluation and Research

**DOI:** 10.3389/fmed.2017.00071

**Published:** 2017-06-12

**Authors:** Marc Kusinitz, Emily Braunstein, Carolyn A. Wilson

**Affiliations:** ^1^CBER, FDA, Silver Spring, MD, United States

**Keywords:** regulatory science, biologics, vaccines, blood safety, cell and gene therapy, clinical trial design, bioinformatics, plasma proteins

## Abstract

Center for Biologics Evaluation and Research enhances and supports regulatory decision-making and policy development. This work contributes to our regulatory mission, advances medical product development, and supports Food and Drug Administration’s regulatory response to public health crises. This review presents some examples of our diverse scientific work undertaken in recent years to support our regulatory and public health mission.

## Introduction

The Center for Biologics Evaluation and Research (CBER) at the Food and Drug Administration (FDA) has regulatory oversight of blood, tissues, and complex medical products that include vaccines, allergenic products, blood-derived products, certain diagnostics and devices, live biotherapeutics, and novel medical products, such as stem cell-derived products, and other cell and gene therapies.

The complex biological products regulated by CBER present a variety of challenges:

(1) The challenge of ensuring product sterility, i.e., the absence of unwanted infectious agents, is compounded with the use of biological materials (e.g., cells, viruses, and bacteria) in the manufacture of biological products. Many of the products that CBER regulates comprise components of, or intact, biologically active cells, tissues, viruses, or bacteria. In most cases, this means that they cannot be subject to terminal sterilization. Quite often, other means of inactivating or removing potentially contaminating infectious agents also cannot be applied without compromising the biological activity of the product.
(2) There may not be a clear strategy for characterizing a manufactured product to verify that it is safe and potent before exposing study subjects in investigational clinical trials. Biological materials are generally not amenable to the type of straightforward analytical evaluation that might be used with a chemical drug for which the molecular structure is known and can be verified. Biological products by their very nature are complex mixtures.
(3) Biological products often elicit immune responses that, depending on the product, might be a desired outcome of the intervention (i.e., vaccine-induced protective immune response) or an unwanted outcome (i.e., immunogenicity of a recombinant protein, cell, or gene therapy). Preclinical models might not predict these effects in humans.
(4) The product’s mechanism of action might not be sufficiently understood to identify appropriate biomarkers of clinical effect and/or to develop appropriate potency assays.
(5) Clinical trial designs need to optimize data collection that enables regulatory evaluation of safety and efficacy. Design must balance the desire to reduce the time and cost of product development with collection of sufficient data, while incorporating patient input and “real world evidence,” as feasible and relevant. These issues are particularly difficult when conducting clinical trials of novel treatments for rare disease where the available pool of patients, and thus the clinical trial group sizes, is quite small.
(6) Evaluation of biological product safety and efficacy does not stop at licensure. Rather, it requires the development of means to accurately identify those post-licensure safety signals that might not have been apparent during clinical investigation, while also collecting additional supporting data on efficacy.
(7) The products within CBER’s regulatory portfolio are often used to defend the public against emerging infectious disease (EID) outbreaks or acts of bioterrorism or burn/blast events. When addressing the threat of an EID, CBER must evaluate the risks to protect and maintain a robust and safe blood supply. CBER must also identify and develop tools to facilitate development and rapid deployment of promising investigational vaccines.
(8) Often the medical products for responding to EID or acts of bioterrorism cannot be ethically or practically evaluated in human clinical trials for efficacy, for ethical or practical reasons. This necessitates development of robust preclinical models for efficacy evaluation.

To meet these challenges, CBER relies on the best available science in its application of relevant laws through regulations and guidance. CBER engages the expertise and ingenuity of its intramural researcher–reviewers to identify and address major scientific gaps. The knowledge acquired facilitates regulatory evaluation and thus fosters development of innovative medical products. The outcomes of these efforts are made available to the public and product developers by publishing the results in peer-reviewed journals. In addition, CBER incorporates knowledge derived from its intramural research program into both informal and formal guidance to sponsors, other policy and regulatory decisions, and development of reference materials and standards to facilitate product evaluation by product developers.

Center for Biologics Evaluation and Research supports the science-based regulation of this diverse and complex product portfolio through a similarly diverse applied research program. The aim of this program is to fill knowledge gaps and develop more effective tools for regulatory decision-making and policy development. The unique perspective of scientists within this regulatory agency facilitates solutions to scientific problems that might benefit an entire class of products, rather than a specific product developed by a single sponsor.

Center for Biologics Evaluation and Research facilitates successful translation of early-stage medical product development into licensed safe and effective therapies. The results help to improve product manufacturing and identifying potential adverse events in marketed drugs and devices.

Center for Biologics Evaluation and Research has four major research goals through which they advance the scientific basis for regulation of biologics, human tissues, and blood.

Here, we provide recent examples from our diverse portfolio of research to demonstrate how CBER’s research contributes to each goal.

## Developing and Evaluating Technology, Reagents, and Standards to Inform and Improve Chemistry, Manufacturing, and Controls

Center for Biologics Evaluation and Research regulators must review the processes used in the manufacture of biologics and critically evaluate their quality and consistency, to ensure that they meet defined standards of safety, purity, and potency (CFR, Title 21 Part 601.2). This requires the use of validated methods and standards for evaluating products, including in-process and lot release tests.

Center for Biologics Evaluation and Research develops new methods and models to evaluate regulated medical products, evaluates or improves existing methods, and develops reagents, such as reference materials for assessing the reproducibility, sensitivity, and specificity of these test methods. The following examples illustrate our efforts to develop and evaluate models, methods, and reference materials and standards.

### Detecting Contaminants in Biological Products

Many biological medical products are live viruses, bacteria, or mammalian cells. This means they are labile, and therefore, cannot be subjected to processes used for traditional biotechnology products, such as pathogen inactivation or removal; nor can they be subjected to terminal sterilization. Therefore, the raw materials and the products themselves must be thoroughly tested to ensure absence of contaminating infectious agents (adventitious agents). CBER scientists evaluated a technique for improving the sensitivity of detecting adventitious viruses. The technique, called tangential flow filtration, concentrates viruses from large volumes of cell substrate as a preliminary step before testing. The use of tangential flow filtration increases the ability to detect low level contaminants (1) and can be used by manufacturers to reduce the risk of contaminating infectious agents.

Center for Biologics Evaluation and Research regulates live biotherapeutic products when they are used to treat a specific condition. One of the challenges of determining the safety and purity of these products is that the live bacteria intended to have a beneficial therapeutic effect can interfere with testing for pathogenic contaminating microorganisms. To address this problem, CBER scientists developed a novel approach that uses viruses (bacteriophages) to selectively target and kill biotherapeutic bacteria in test samples, allowing unwanted pathogenic microbes to grow unimpeded. This enables use of standard laboratory methods to detect the harmful contaminants (2).

### Improving Quality and Consistency of Biological Products

Many of CBER’s products are complex and difficult to characterize. In fact, the critical attributes that correlate with safety, purity, and potency may not be known. Stem-cell-based therapies are an emerging class of products that are particularly challenging in this regard. Stem cell research holds the promise of innovative medical treatments for a variety of diseases, but also poses unique challenges to regulatory decision-making.

One major challenge facing regulators and industry is that we do not know the key, measurable properties of stem cells that correlate with specific, desired clinical outcomes. Several years ago, CBER assembled a consortium of scientists charged with addressing this challenge. The consortium uses different methods in an effort to identify critical stem cell attributes. In addition to evaluating some standard approaches, such as traditional flow cytometry for cell surface markers, CBER scientists are also developing new methods and using technological approaches such as proteomics, single-cell RT-PCR, epigenetics, and microarray.

To interpret the results of characterization assays, CBER is also developing ways to correlate assay results with functional assay outcomes, such as *in vivo* or *in vitro* models of wound repair, quantitative differentiation capacity, or immunosuppression (3–9). A recent example of the work being performed by the consortium is the development of a new method to predict differentiated functional capacity of human mesenchymal stem cells (multipotent mesenchymal stromal cells) after osteogenic induction. Using high-dimensional, single-cell morphological information, CBER scientists demonstrated that within 3 days, the morphological data correlated with 35-day mineralization assays with >90% accuracy (8). The development, evaluation, and communication of innovative methods, such as the one described above to identify bone differentiation capacity, provide a new stem cell-based tool to the community to facilitate development of innovative medical products.

### Reference Materials and Standards

During an outbreak of an emerging transfusion-transmitted infectious disease (TTID), there is a delicate balance of priorities between maintaining the availability of blood for life-saving transfusions and reducing or eliminating the risk of TTID. In such situations, CBER uses a multipronged strategy to facilitate the complex decision-making required to ensure the availability of safe blood:

- Holding public workshops to discuss risk assessment for a given EID for blood and blood products (10)
- Developing new tools (e.g., modeling) to assess EID rates and risks
- Developing reference materials to facilitate evaluation of new assays to screen for EIDs.

In the last 15 years, the safety of the blood supply has been threatened by the agents of transmissible spongiform encephalopathy including variant Creutzfeldt–Jakob disease, as well as emerging and re-merging viruses and other pathogens: West Nile virus, chikungunya virus (ChikV), *Babesia microti*, dengue virus, and most recently, Ebola and Zika viruses.

Center for Biologics Evaluation and Research develops RNA reference standards that sponsors use to evaluate the sensitivity, specificity, and reproducibility of their nucleic acid-based tests intended for clinical diagnosis or blood screening for emerging and reemerging TT viruses. CBER typically characterizes these reference materials through multi-laboratory studies, such as those recently performed for West Nile virus (11), dengue virus (12), and ChikV (13). CBER then distributes these reference materials as CBER National Reference Reagents. CBER is currently developing reference standards to use in screening blood or tissue for transmissible spongiform encephalopathy (TSE) agents (14).

In spring 2016, CBER participated in a coordinated public health response to the emerging Zika virus outbreak. CBER scientists developed and coordinated a multi-laboratory effort to generate Zika virus RNA reference materials that were adopted by the World Health Organization. Other international standards organizations, such as the National Institute for Biological Standards and Control, have also adopted and distributed them worldwide.

In addition to protecting the blood supply from the risk of TTIDs, CBER laboratories actively support the development and lot release testing of seasonal and pandemic influenza vaccines in many ways (15):

(1) Early serology testing of circulating strains to identify strain prevalence
(2) Generation of candidate vaccine strains
(3) Generation of reagents that are distributed to manufacturers for lot release testing
(4) Independent verification of lot release testing results.

Center for Biologics Evaluation and Research prepares reagents to test influenza vaccine potency and ensure standardization of vaccine quality made by manufacturers. Traditionally, the vaccine strain is grown in eggs; bromelain is then used to purify the HA, which is used to immunize sheep. This technique is successful with most virus strains. However, in 2013, when novel H7N9 viruses emerged in China, the Center faced a major, unexpected bottleneck in preparing vaccine reagents when the traditional approach yielded substandard results. CBER scientists overcame that bottleneck by expressing H7N9 HA glycoprotein as a virus-like particle (VLP) using a vaccinia vector—a technique previously developed in our laboratories. Boosting sheep with the VLPs instead of the traditional HA yielded antiserum that significantly improved the quality of lot release assay results (16).

This type of innovation at CBER enables timely reagent production and might overcome future potential bottlenecks in influenza vaccine production that have occurred in the past for both seasonal and pandemic strains.

## Developing and Assessing Non-Clinical Models and Methods Predictive of Clinical Performance with Respect to Toxicity and Effectiveness

Food and Drug Administration relies on data from non-clinical models and methods to support regulatory decisions on first-in-human clinical trials. In particular, before human, phase 1 clinical studies are initiated, the primary focus of FDA review is on safety. Non-clinical studies may identify candidates that should not proceed to human clinical trials. However, there are products for which issues are identified only after they progress into clinical testing, or even wider scale use on the market (for example, see the US FDA Report: 22 case studies that show divergent results between phase 2 and phase 3 trials^1^). These observations, combined with the finding that many products also fail in clinical trials due to lack of efficacy, suggest there is a need for improved non-clinical models and methods that more reliably predict product performance in humans. For the purpose of this discussion, non-clinical models and methods include *in silico* predictive models, as well as *in vitro* assays and *in vivo* animal models.

### Learning from Medical Products That Fail in Clinical Trials

Immune responses to therapeutic protein products can compromise their therapeutic effects and trigger adverse events, some of which (e.g., anaphylaxis) can be life threatening. To ensure product developers consider this risk, FDA issued guidance for industry (“Immunogenicity Assessment for Therapeutic Products”). The guidance—a collaboration between CBER and Center for Drugs Evaluation and Research (CDER)—incorporates knowledge and improved mechanistic understanding gained from clinical trials, post-marketing adverse events, and laboratory and *in silico* studies, including CBER research (17–19).

The studies that informed the guidance continue today, providing improved mechanistic insights that lead to new ways to estimate risks of immunogenicity posed by recombinant protein products. Recently, CBER scientists began using a bioengineered analog of the clotting Factor VIIa to identify how neo-sequences introduced into protein drug analogs might induce development of antidrug antibodies (ADA). The work includes developing an approach to identify patients most likely to be affected by this adverse event. ADA impair the drug’s therapeutic benefit and can also be life-threatening themselves. Therefore, improved methods to predict development of ADA can enhance safety and efficacy of innovative medical products.

In this case, the drug, vatreptacog alfa, had three minor changes engineered into its structure to improve its efficacy. While the parent drug had been used for two decades without reports of ADAs, the analog triggered ADAs during a Phase 3 clinical trial, ending its development. Using computational and experimental methods, CBER researchers found that in all 11 patients who had ADAs to vatreptacog alfa peptides, there was at least one HLA class II protein that was both predicted and then shown *in vitro* to bind avidly to one of the peptides with the same mutations as found in the analog. However, only 44% of patients without ADAs had an HLA allele specific for peptides from vatreptacog alfa (20).

This approach to analysis of bioengineered protein drugs and the patient characteristics that impact development of ADA could inform application of precision medicine to identify patients likely to benefit from the products without the risks of developing ADAs. With improved predictive tools, the resulting clinical trials of such products might become less costly and more efficient.

### *In Vitro* and Animal Models Identify Mechanisms of Product Toxicity or Reduced Efficacy; Outcomes Guide Future Product Development

Adenovirus vectors are often used in clinical trials of gene therapy because they possess two major desired traits:

- Ability to be manufactured and purified to high concentration and purity
- High expression levels of the potentially therapeutic protein in host cells

In spite of these positive traits, clinical development of adenovirus vector-mediated gene therapy has been hampered by the low targeting efficiency of vectors.

Center for Biologics Evaluation and Research scientists are combining animal model and *in vitro* studies to investigate how the vector interacts with the immune system. They made the novel observation that adenovirus vectors of serotype 5 (Ad5, the first serotype used in gene therapy clinical trials) forms complexes with the coagulation factor X (FX) that shield the vector from antibodies and complement. This shield facilitates vector transduction of hepatocytes. In contrast, vectors unable to form complexes with FX showed significantly lower levels of infection of and gene expression in, the liver and lung (21).

The researchers subsequently showed that replacing the FX shield with a polyethylene glycol shield prevented macrophages from neutralizing the vector *in vitro* and demonstrated significantly improved transduction in murine models (22). These findings suggest that such shielding could greatly improve the clinical efficiency of Ad5 in human gene therapies.

Center for Biologics Evaluation and Research also develops and uses animal models to evaluate vaccines for safety and effectiveness. For example, in the face of a resurgence of pertussis (whooping cough) in the past decade, CBER developed a baboon model. The model is being used for studies aimed at improving the safety and effectiveness of pertussis vaccines.

Following introduction of the whole-cell pertussis vaccine in the 1950s, the occurrence of pertussis in the US decreased by over 90%. However, whole-cell pertussis vaccine caused soreness at the injection site and fevers in children (described as reactogenicity). Therefore, the US replaced whole-cell pertussis vaccine with the acellular pertussis (ACP) vaccine in 1997. Subsequently, there was a steady rise in cases, which peaked at nearly 50,000 in 2012, according to the Centers for Disease Control and Prevention.^2^

To investigate the cause of this rising trend of pertussis cases in the face of ACP vaccination, CBER developed the first animal model to recapitulate the disease and provide scientific evidence of airborne transmission (23). Studies with this baboon model demonstrated that third-trimester maternal vaccination with ACP protected neonates from disease when challenged at 5 weeks of age (24). However, even in the absence of disease, vaccinated animals showed evidence of bacterial infection, colonization, and ability to transmit to vaccinated animals (25).

Additional studies using the baboon model showed significant differences in the type of immune responses to natural infection, whole-cell vaccine, and ACP. Specifically, unlike the ACP vaccine, any of the three whole-cell vaccines currently approved for use outside the US significantly accelerated the clearance of the causative organism, *Bordetella pertussis*, from the nose and throat. Moreover, whole-cell vaccines boosted production of the immune system protein IL-17, which suggests this molecule plays a role in clearing the infection (26).

This finding is important because it provides the scientific basis for improving the pertussis vaccine: the use of novel adjuvants to boost Th17 responses to ACP vaccines. Thus, this work provides a roadmap for designing the next generation of pertussis vaccine. The goal is to develop a vaccine that has low reactogenicity but retains the protective immune responses that are associated with protection against infection and disease.

## Improving Clinical Evaluation Pre- and Post-Licensure through Use of Big Data, Innovative Designs, and Statistical, Analytical, and Modeling Approaches

A large study of clinical development programs between 2006 and 2015 found that the likelihood that a drug candidate would progress from phase 1 through licensure was 9.6%. Some of the factors that impacted success rates were patient population and selection, especially in rare disease studies, as well as clinical trial complexity in large heterogeneous patient populations (27). These observations underscore the need to improve clinical trial designs.

### Improving Clinical Trial Design Pre-Licensure

Similar to the larger study from Bio, Biomedtracker, and Amplion, CBER studied the problems seen in regulated clinical trials, focusing initially on adaptive designs, i.e., clinical trials that enable knowledge gained during the research to affect ongoing conduct or analysis of the trial. We subsequently published a 6-year analysis (2008–2013) of adaptive design proposals from product sponsors. The paper provides recommendations to sponsors for developing such proposals, with the aim of encouraging optimal design (28).

In another project, CBER statisticians used a novel version of a Bayesian two-stage adaptive design to analyze a hypothetical Phase II trial of immune globulin (intravenous; IGIV)—an antibody-based product used as replacement therapy in patients with humoral immunodeficiencies. The study confirmed the validity of using this statistical technique to end a clinical trial early due to either treatment futility or efficacy, an approach that can save developers of novel medical products both time and money (29).

### Developing New Approaches to Identify Post-Licensure Safety Signals

Center for Biologics Evaluation and Research also works to improve safety surveillance using large databases, such as the US Vaccine Adverse Event Reporting System and the FDA Adverse Event Reporting System for drugs. Optimal use of these databases requires automated retrieval of information useful for reviewer analysis. The current natural language processing systems are often hampered by low quality text (e.g., poor grammar, many abbreviations). CBER developed an algorithm for analyzing low quality texts that is minimally dependent on grammatical and syntactic information (30). This will speed reviewer extraction and analysis of important information, and accelerate recommendations about post-marketing adverse events related to medical product use. It might also contribute to future research in text mining of electronic health records.

Additionally, a major limitation to timely tracking of vaccine adverse events using reporting databases, such as those used in the FDA’s Sentinel System, is that the most recent data are 6–9 months old. While these “mature” data give a broad, long-term view of events, it does not allow for timely regulatory responses to adverse events. CBER developed a way to access, use, and evaluate “fresh” data for timely influenza vaccine safety surveillance in the Sentinel System. This approach is now being used to develop an infrastructure that works for other FDA-regulated medical products, such as blood products, that require faster access to safety information and a review response that is closer to “real-time.” (31).

## Preparing for Future Regulatory and Public Health Challenges

Changes in science and technology are occurring rapidly, impacting many of the products CBER regulates. This means that the Center’s research program must evolve and anticipate, when feasible, developments that are likely to yield innovative medical products.

As resources permit, CBER expands capabilities in key technologies, such as high resolution analytical instrumentation (NMR and mass spectrometry), “next-generation sequencing” (NGS), multiparameter analytic and sorting flow cytometry, high resolution microimaging of fixed and living cells and tissues, and tools for sequential imaging of living animals, such as digital X-ray, ultrasound, and a 4.7 T MRI.

In addition, CBER developed hardware infrastructure and a bioinformatics team to support transfer, storage, and analysis of large, complex datasets. This high-performance integrated virtual environment (HIVE) supports analysis of NGS data generated not only by our own researchers but also provided by sponsors in regulatory submissions. In addition, CBER is adopting the advanced computational platform provided by HIVE for analysis of other complex data, such as high resolution mass spectra, and integration of product, preclinical, and clinical data. These new tools enable improved regulatory evaluation of medical products. The underlying code supporting HIVE is open source,^3^ and the team of HIVE developers and research scientists using HIVE publish the tools, applications, and results of using HIVE (32–40).

To keep pace with new technology and to anticipate innovative product development, CBER needs scientists with state-of-the-art knowledge. CBER recruits scientists to develop and establish new research programs that respond to emerging public health challenges, new technology, and regulatory needs. New programs that have been initiated in the past 3 years include the following:

- Vaccine development for emerging pathogens (e.g., norovirus)
- Using the microbiome to treat infection with antibiotic-resistant bacterial pathogens
- Using microphysiologic systems to improve safety and effectiveness of tissue-engineered medical products
- Mechanistic studies of cellular differentiation of induced pluripotent stem cells (iPSCs)
- Effects of the gene editing endonucleases (i.e., Cas9) on genome integrity and function of human iPSCs
- Factors that influence T cell activation and how that might inform evaluation of CAR-T cell-based therapies (chimeric antigen receptor)

## Conclusion

Center for Biologics Evaluation and Research makes critical contributions to FDA’s efforts to fulfill its regulatory mission. The Center’s active intramural research program provides three important capabilities: (1) rapid responses to urgent public health needs, such as a newly identified post-marketing safety signal or EID outbreak; (2) development and evaluation of new methods to advance development of products by providing better tools for evaluating safety, effectiveness, or product quality; (3) preparation for future products by evaluating potential safety concerns or developing new methods and models to characterize complex new products that enhance effectiveness and product quality.

Center for Biologics Evaluation and Research’s scientists play a dual role: they not only perform applied research relevant to key regulatory needs but also participate in the review of submitted regulatory files, perform inspections, participate and organize timely advisory committees and workshops, and inform development of guidance and regulatory decision-making. This dual role of the researcher–reviewer allows CBER to rapidly incorporate the findings of our research program into daily informal guidance provided to sponsors. It also enables the center to share the outcome of our research with both the public and the scientific and regulated communities by publishing in peer-reviewed scientific journals and presenting our research findings at public workshops, advisory committees, and relevant scientific and professional meetings. Essentially, the capabilities resulting from an active intramural research program enable CBER to be prepared, flexible, proactive, and responsive in support of its regulatory mission and its efforts to facilitate development of safer and more effective innovative medical products.

## Author Contributions

MK and EB: provided input into the overall organization, content, and approach for the article. Provided substantive input into the draft of the article at each stage. Wrote initial drafts of some sections of the article. CW: primary author who wrote the initial draft of the article, coordinating input from coauthors, and finalized the article for submission.

## Conflict of Interest Statement

The authors declare that the research was conducted in the absence of any commercial or financial relationships that could be construed as a potential conflict of interest.

## Footnotes

^1^https://www.fda.gov/aboutfda/reportsmanualsforms/reports/ucm535541.htm.

^2^https://www.cdc.gov/pertussis/fast-facts.html.

^3^https://github.com/FDA/fda-hive.
